# Importance of Regulating the Teeth, and Employment of Gum Elastic Rings in the Operation

**Published:** 1850-10

**Authors:** E. G. Tucker

**Affiliations:** Boston


### ARTICLE VI.

### *Importance of Regulating the Teeth, and Employment of Gum-Elastic Rings in the Operation.*

Dr. C. A. Harris:

*Dear Sir:*—I shall not readily forget our last meeting at Saratoga, where so commendable a feeling prevailed for advancing and elevating our profession.

The dentist is looked upon too much as a secondary character—one whose claims rest rather upon accident than legitimate science. This is an error; and yet it is an error easily explained. For a long time the teeth were neglected, and the first operators were doubtless mere mechanics. The surgeon was called upon if a tooth was to be extracted, but the idea of *repairing* the ravages of disease in these organs, is comparatively modern.

Perhaps the physical constitution in former times was more perfect, and less liable to disease or partial decay, than at present. One is not apt to take any special notice of those parts of the system which occasion no pain; hence it is fair to presume, that no special care was taken of the teeth until they began to fail of their original purpose.

Thus it has been with the diseases of the body. Man has, by his imprudence and ignorance, not only added to the number every succeeding generation, but by his violations of the natural laws, has added to their aggravation; consequently, new labors are constantly before the physician and surgeon, rendering their lives one of perpetual study. How can it be otherwise with the dentist? He is now required to be well read in medical science—indeed to be worthy of a diploma of a medical college.

Whatever pertains to the teeth, whether we view them separately, or as parts of a various whole, comes within the limits of our profession. It is requisite that the dentist should understand the laws of their formation, and the condition of their preservation. In this respect, his services should commence with the child. To prevent disease is far more important than to cure it, both on the score of expense and comfort. This is, perhaps, a truth more applicable to our profession than any other. People cannot scrutinize their own mouths, or the mouths of others, unless they have the necessary knowledge; and hence their teeth may decay beyond *remedy*, or may grow so irregular as to defy the most consummate skill. The removal of a tooth in the child may secure regularity, prevent premature decay, and thus ward off the pain and destruction that is sure to visit the adult tooth.

Parents, therefore, cannot begin too soon to watch the growth of their children's teeth, and to seek the aid of the dentist. They should realise that this advice is not given for the purpose of increasing the labors of the dentist, but to lessen them. If he does more for the *girl*, he will be called upon to do less for the *lady;* and if he performs his duty to *the boy*, there may be no occasion for calling him to the *man*.

The business of regulating teeth is new to most people, and yet, when we consider to what expense some are willing to go for comfort, convenience and appearance, it must become, in time, a very important branch of our profession.

But few have thought it practicable to alter what nature has done in the *arrangement of our teeth;* in fact, but few have given to the subject any consideration whatever. In every thing pertaining to the teeth, the dentist should have as full control as the physician has with the body of his patient. The child should be submitted to his judgment and to his skill. What he advises should be executed with the same promptness as the prescription of the physician.

I have been led to suppose that something might be contrived as a substitute for spiral springs, gold bands, &c. I have made, for experiment, rubber tubes, of different sizes and thicknesses, so that rings may be cut of any dimensions. No substance that I can think of, would be more likely to prove pleasant to the mouth, or less likely to irritate the tender parts; besides, by these tubes, we may have a great variety of means, *as to power*, in the simplest and cheapest form. We can commence with a small force and increase, or reverse the operation, according to the circumstances of the case.

The frame-work, made of silver or gold, may be prepared with hooks or clasps at different parts of the plate, so as to attach the loops with ease, by the use of two instruments, such as pluggers, stretching from tooth to tooth, whether near or remote. It is easy to conceive of a variety of operations with these *pulling rings*, and *to you* it is quite unnecessary to enlarge.

I only hope that the contrivance may meet with your approval. If it proves to be useful, I shall have great satisfaction in the belief that I have, in the smallest degree, contributed my mite to the stock of practical knowledge.

With much respect and esteem,

I remain your friend,

E. G. TUCKER.

Boston, *October* 3*d*, 1850,
*No* 4 *Hamilton Place.*

